# Reply to Moossavi and Azad, “Quantifying and Interpreting the Association between Early-Life Gut Microbiota Composition and Childhood Obesity”

**DOI:** 10.1128/mBio.00047-19

**Published:** 2019-02-12

**Authors:** Maggie A. Stanislawski, Dana Dabelea, Brandie D. Wagner, Nina Iszatt, Cecilie Dahl, Marci K. Sontag, Rob Knight, Catherine A. Lozupone, Merete Eggesbø

**Affiliations:** a Department of Epidemiology, Colorado School of Public Health, Aurora, Colorado, USA; b Department of Pediatrics, University of Colorado School of Medicine, Aurora, Colorado, USA; c Department of Biostatistics and Informatics, Colorado School of Public Health, Aurora, Colorado, USA; d Department of Environmental Exposure and Epidemiology, Norwegian Institute of Public Health, Oslo, Norway; e Department of Community Medicine and Global Health, University of Oslo, Oslo, Norway; f Center for Public Health Innovation, CI International, Littleton, Colorado, USA; g Department of Pediatrics, University of California San Diego, La Jolla, California, USA; h Department of Computer Science and Engineering, University of California San Diego, La Jolla, California, USA; i Center for Microbiome Innovation, University of California San Diego, La Jolla, California, USA; j Department of Biomedical Informatics and Personalized Medicine, University of Colorado School of Medicine, Aurora, Colorado, USA; k Lifecourse Epidemiology of Adiposity and Diabetes (LEAD) Center, Aurora, Colorado, USA; Yale School of Public Health

**Keywords:** microbiome, obesity, pediatric, molecular epidemiology

## REPLY

First, we thank Drs. Moossavi and Azad for their thoughtful comments about our recent paper (1). We welcome the opportunity to clarify some of our findings and the associated caveats. The authors note that it seems biologically implausible that 50% of BMI variation in childhood might be explained by gut microbiota during infancy. In our paper, we tried to emphasize both the limitations of our methods and the uniqueness of the cohort, which together might lead to high estimates for *R*^2^. Methodologically, we used the same cohort for variable selection, model training and testing because the sample size was rather small to explicitly split the data into training and test sets. We used repeated cross validation for our estimates of *R*^2^ in order to compensate for this choice, but this method might still lead to an overestimate of model fit (2). While there are numerous well-designed studies of the infant gut microbiota, including the CHILD study, NoMIC is fairly unique in the number and timing of samples collected during childhood and in the homogeneity of the cohort population, creating challenges for external validation. While the numeric values of *R*^2^ might prove to be high relative to those for other cohorts, and the specific taxa predictive of later BMI may differ, we believe that our main findings are robust: (i) that the gut microbiota in infancy is associated with later BMI; (ii) that this association is present in the first days/months of life, before other contributors (such as food intake) are present; and (iii) that the association becomes stronger at 1 year than during the first 4 months of life and even stronger at 2 years. We note that one recent study found that the contribution of the gut microbiome toward explaining variation under many conditions, including BMI, was greater than that of genetics (3). We expect that the association between gut microbiota in infancy and later BMI will show high variability across populations, just as the strengths of genetic associations vary (3, 4), depending on genetic background and other cohort characteristics. We welcome collaboration with other cohorts, including the CHILD study, in order to examine the consistency of these patterns across studies.

We extensively discussed the relations among early-life exposures, gut microbiota, and later adiposity in order to define the conceptual frameworks and causal pathways presented in the paper, and we agree that these relations are complex and could be approached differently. We did not control for factors that come into play after the time points at which the gut microbiotas were sampled because these factors would not meet the definition of a confounding variable, and we did not control for maternal obesity because we were specifically interested in this pathway to childhood obesity. However, we did examine the amount of variation explained by possible determinants of childhood BMI, which ranged from 0.03% to 7.88% in simple linear-regression models of childhood BMI z-scores (Table 1). A multiple-linear-regression model including all of these predictors had an *R*^2^ value of 28.3% and an adjusted *R*^2^ of 19.5%, which is much higher than seen in the CHILD cohort. The gut microbiota is likely partly a reflection of some of these environmental factors, particularly gestational age, delivery mode, and breastfeeding. The *R*^2^ values in the random forests with and without the inclusion of confounding variables were very similar (1), supporting the idea that the variation in BMI explained by these exposures is captured to some extent by these gut microbiota taxa.

**TABLE 1:** Results of simple linear-regression models of childhood BMI z-score as a function of each of the predictors listed in the NoMIC cohort^*a*^

Parameter	β estimate	*P* value	*R*^2^ (%)
Maternal overweight/obesity	0.56	<0.001	7.28
Excessive maternal GWG	0.54	0.001	7.88
Maternal BMI	0.06	<0.001	7.76
Child sex	0.18	0.254	0.80
Birth wt (kg)	0.26	0.006	4.52
Duration of exclusive breastfeeding	–0.07	0.041	2.55
Delivery mode	0.18	0.295	0.67
Gestational age	0.04	0.07	2.00
Twin status	–0.64	0.007	4.32
Antibiotic exposure in first 30 days	–0.05	0.833	0.03
Parity	–0.55	0.012	4.20
Norwegian ethnicity	0.09	0.754	0.06
Maternal education	–0.24	0.058	2.18
Maternal smoking	0.35	0.01	3.95

a The NoMIC cohort had 165 participants. GWG, gestational weight gain.

We did not see any preliminary evidence of an association between sex and the infant gut microbiota in this cohort or of sex differences in the relation between maternal obesity and infant gut microbiota. We agree that breastfeeding (and breast milk composition) might modify the relation between maternal BMI and infant gut microbiota and hope to examine this more fully in future work. However, our preliminary analyses did not support a strong interaction. We used permutational analysis of variance (ANOVA) in R (5, 6) in order to assess whether there was a significant interaction between maternal overweight/obesity status and the duration of exclusive breastfeeding in relation to overall infant gut microbiota composition, and it was generally not significant (*P* values > 0.2). The one possible exception was for the gut microbiota at 1 year, at which time the interaction term had a *P* value of 0.056 (uncorrected for multiple comparisons).

Again, we thank the authors for their helpful comments. We agree that this type of scientific dialogue is beneficial in order to strengthen our understanding of these complex relationships and to generate ideas for future work and collaborations.
